# Author Correction: Molecular mechanisms of receptor recognition and antibody neutralization of coxsackievirus A6

**DOI:** 10.1038/s41467-026-75441-7

**Published:** 2026-07-16

**Authors:** Xianliang Ke, Xue Li, Zeyu Liu, Kexin Liu, Weichi Liu, Xingyu Yan, Bo Shu, Chao Zhang

**Affiliations:** 1 State Key Laboratory of Virology and Biosafety, Wuhan Institute of Virology, Center for Biosafety Mega-Science, Chinese Academy of Sciences, Wuhan, Hubei China; 2 Shanghai Institute of Infectious Disease and Biosecurity, Fudan University, Shanghai, China

Correction to: *Nature Communications* 10.1038/s41467-025-67666-9, published online 18 December 2025

In the original version of this Article, a copy–paste error occurred during the calculation of the IC_50_ values presented in Figure 1, resulting in the loss of the formula when the data were copied from Excel.

This has now been corrected in both the PDF and HTML versions of the Article.

