# Supplementary material for: Molecular mechanisms of receptor recognition and antibody neutralization of coxsackievirus A6
Source: Nat Commun. 2025 Dec 18;17:934. doi: 10.1038/s41467-025-67666-9 (PMC12830800; doi:10.1038/s41467-025-67666-9)
Supplement: Supplementary file 1 — Supplementary Information [file 41467_2025_67666_MOESM1_ESM.pdf]

Supplementary Information for:

Molecular mechanisms of receptor recognition and antibody neutralization of coxsackievirus A6

This Supplementary Information PDF includes:

Supplementary Figures 1-15

Supplementary Tables 1-8

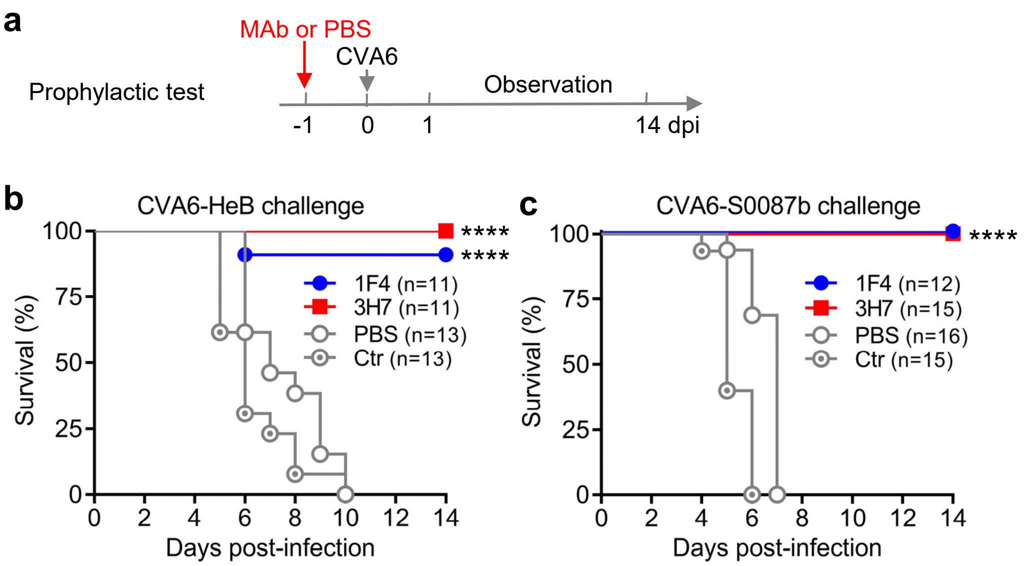

Supplementary Figure 1. Prophylactic efficacy of MAbs 1F4 and 3H7 against CVA6-HeB and CVA6-S0087b lethal infections in mice. Neonatal mice were intraperitoneally administered PBS, 10 μg/g of MAb 1F4, 3H7 or control antibody (an anti-SARS-CoV-2 antibody) 24 hours prior to challenge with CVA6-HeB or CVA6-S0087b. (**a**) Study design schematic. (**b-c**) Survival curves comparing antibody-treated groups with PBS controls. Statistical significance was determined by Log-rank (Mantel-Cox) test. ****, p < 0.0001. The number of mice in each group is shown in parentheses. Source data are provided as a Source Data file.

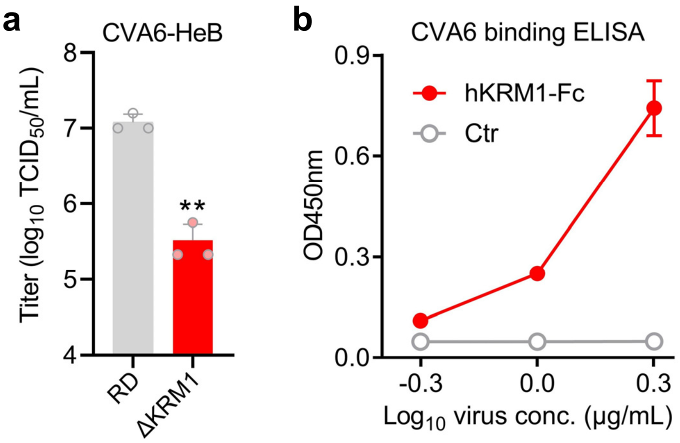

Supplementary Figure 2. KRM1 functions as a critical receptor required for productive CVA6 infection. **(a)** Wild-type RD cells and *KRM1* knockout (Δ*KRM1*) cells were infected with CVA6-HeB, and viral titers were quantified at 24 hours post-infection (hpi). Significance was determined by two-tailed Student's t-test. Data are means ± SD of triplicate biological samples. Each data point represents a single biological replicate. **(b)** The interaction between CVA6 and human KRM1-Fc (hKRM1-Fc) was analyzed by ELISA. Serial dilutions of purified CVA6-HeB virions were immobilized on ELISA plates and probed with hKRM1-Fc or control (ACE2-Fc). Binding signals are expressed as mean optical density (OD) ± SEM from triplicate wells. Source data are provided as a Source Data file.

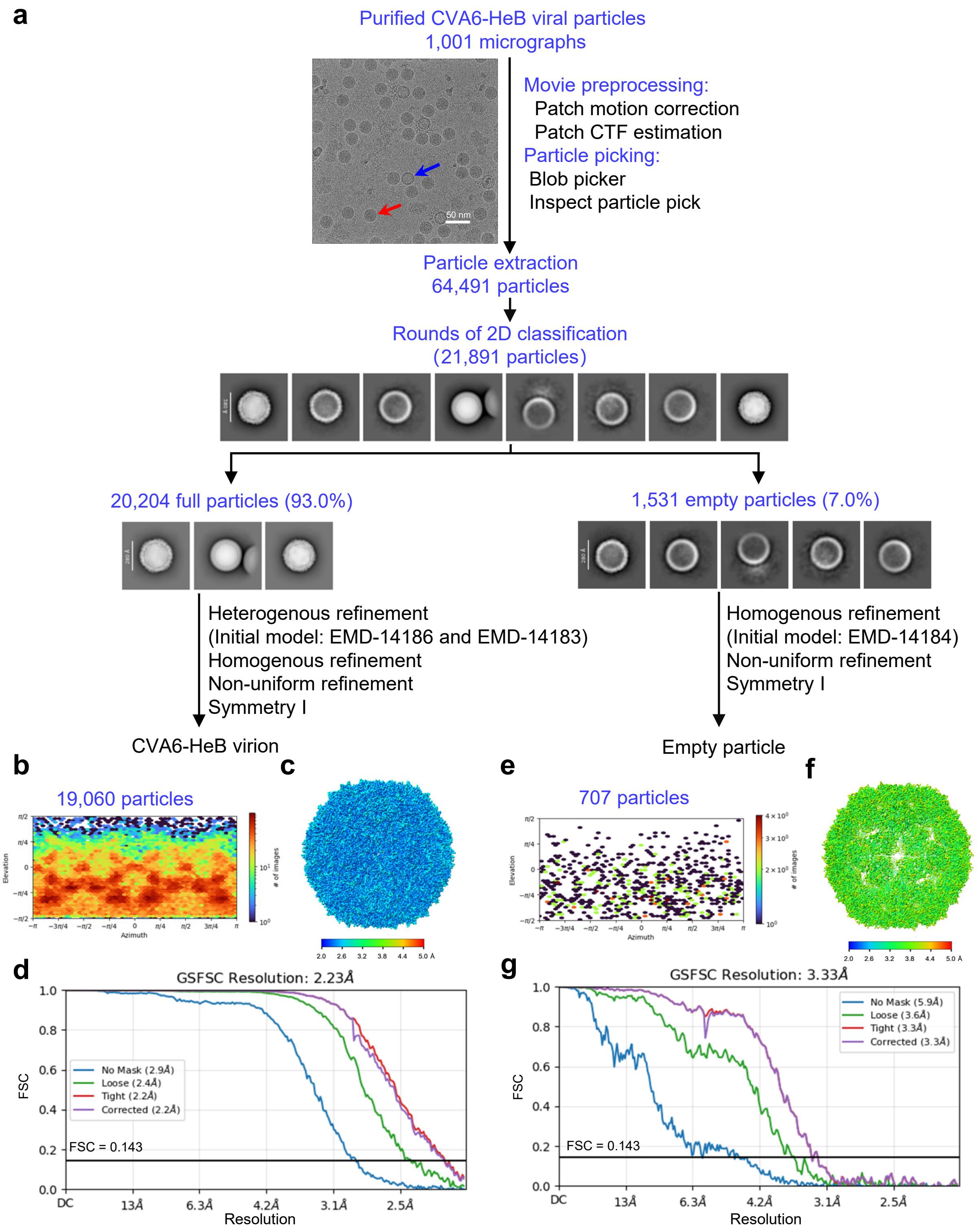

Supplementary Figure 3. Cryo-EM structural analysis of UV-inactivated CVA6-HeB viral particles. (**a**) A representative cryo-EM image of CVA6-HeB viral particles and data processing workflow using cryoSPARC. Red and blue arrows indicate full and empty particles, respectively. The 3D reconstruction utilized previously reported CVA6-Gdula density maps (EMD-14186: mature virion; EMD-14184: empty particle; EMD-14183: A-particle) as initial models. **(b, c, d)** Particle angular distribution, local, and global resolution estimation of mature CVA6 virion. **(e, f, g)** particle angular distribution, local, and global resolution estimation of CVA6 empty particle. Local resolutions were calculated in cryoSPARC (v4.5.3) and visualized in UCSF ChimeraX (v1.9). Global resolutions were determined in cryoSPARC based on the gold-standard Fourier shell correlation (FSC) curve at a cutoff of 0.143.

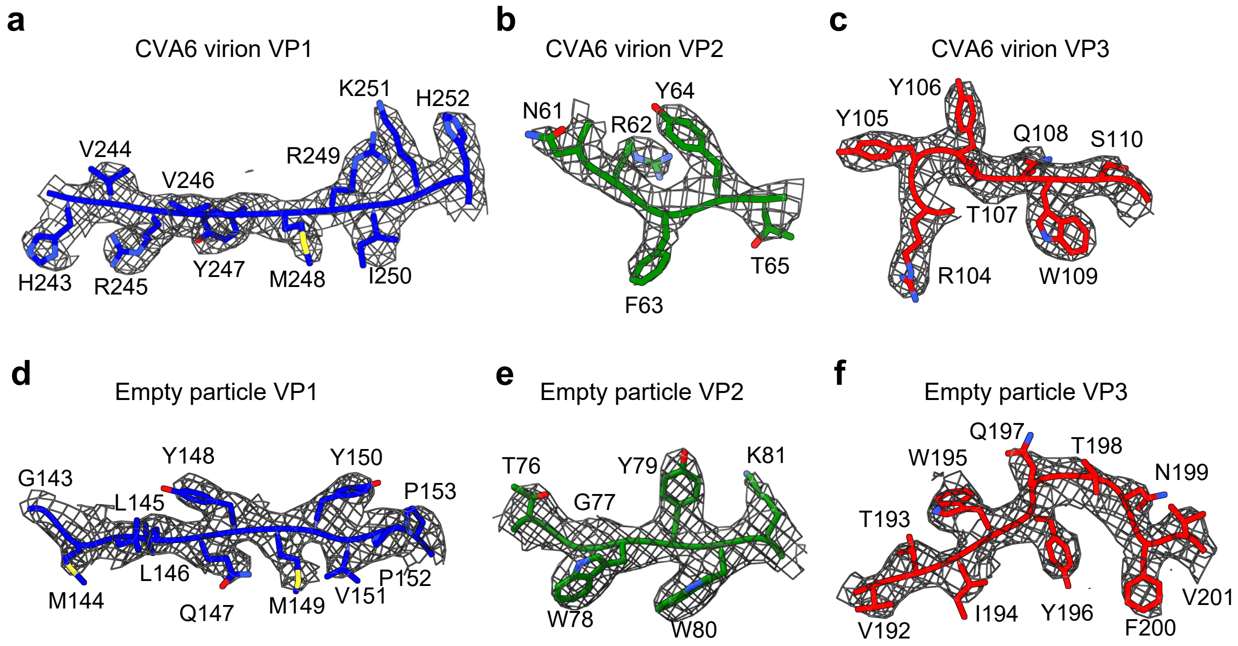

Supplementary Figure 4. Quality of the cryo-EM density maps for CVA6 particles (related to **Figure 3a-b**). Representative structural motifs (colored sticks) are shown fitted into their corresponding density maps (gray mesh). UV-inactivated CVA6 virion: (**a**) VP1 (blue), (**b**) VP2 (green), (**c**) VP3 (red). CVA6 empty particle: (**d**) VP1 (blue), (**e**) VP2 (green), (**f**) VP3 (red).

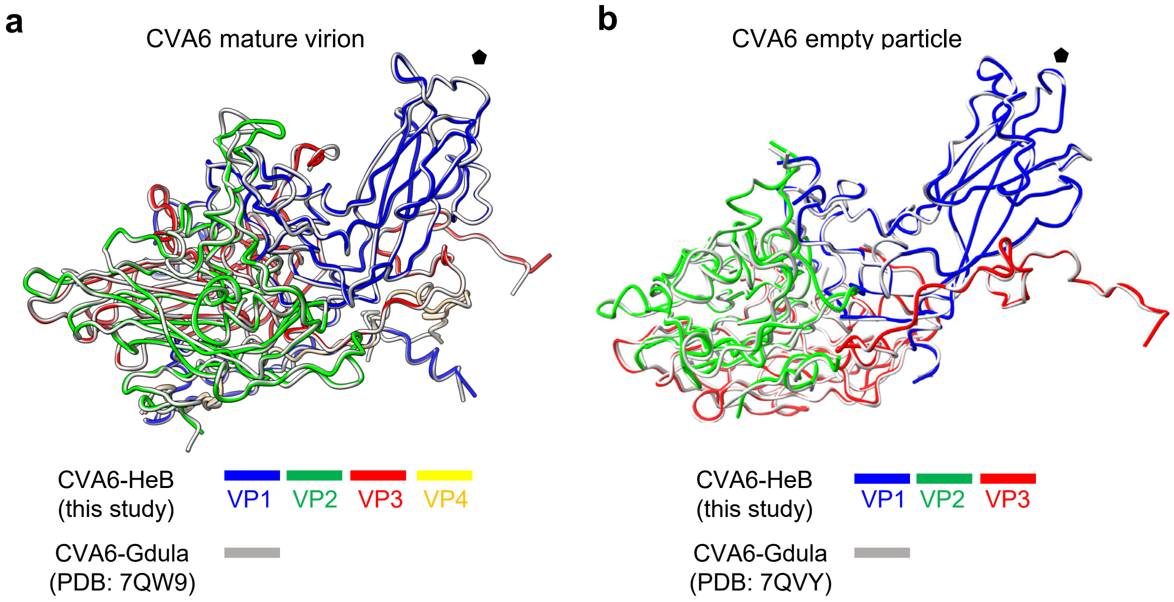

c

Mature virion		VP1	VP2	VP3	VP4	Protomer
CVA6-HeB (this study) vs. CVA6-Gdula (PDB: 7QW9)	Aligned Cα	292 / 292	242/ 249	235 / 240	45 / 45	815/ 826
	RMSD (Å)	0.3	0.3	0.3	0.4	0.3

d

Empty particle		VP1	VP2	VP3	VP4	Protomer
CVA6-HeB (this study) vs. CVA6-Gdula (PDB: 7QVY)	Aligned Cα	212 / 213	206 / 206	216 / 218	-	634 / 638
	RMSD (Å)	0.5	0.5	0.6	-	0.5

Supplementary Figure 5. Structural comparison of CVA6 protomers between the HeB strain (this study) and the prototype Gdula strain. (**a**) Superposition of mature virion protomers from the HeB strain (colored) and the Gdula strain (gray; PDB: 7QW9), shown as ribbon diagrams. Subunits in the HeB protomer are labeled as follows: VP1 (blue), VP2 (green), VP3 (red), and VP4 (yellow). (**b**) Superposition of empty particle protomers from the HeB strain (colored) and the Gdula strain (gray; PDB: 7QVY), rendered as ribbons. (**c**) Root-mean-square deviation (RMSD) analysis of mature virion protomers between the HeB strain and the Gdula strain (PDB: 7QW9). (**d**) RMSD analysis of empty particle protomers between the HeB strain and the Gdula strain (PDB: 7QVY). Structural alignments and RMSD calculations were performed using UCSF ChimeraX. Protomer chains were preprocessed in WinCoot.

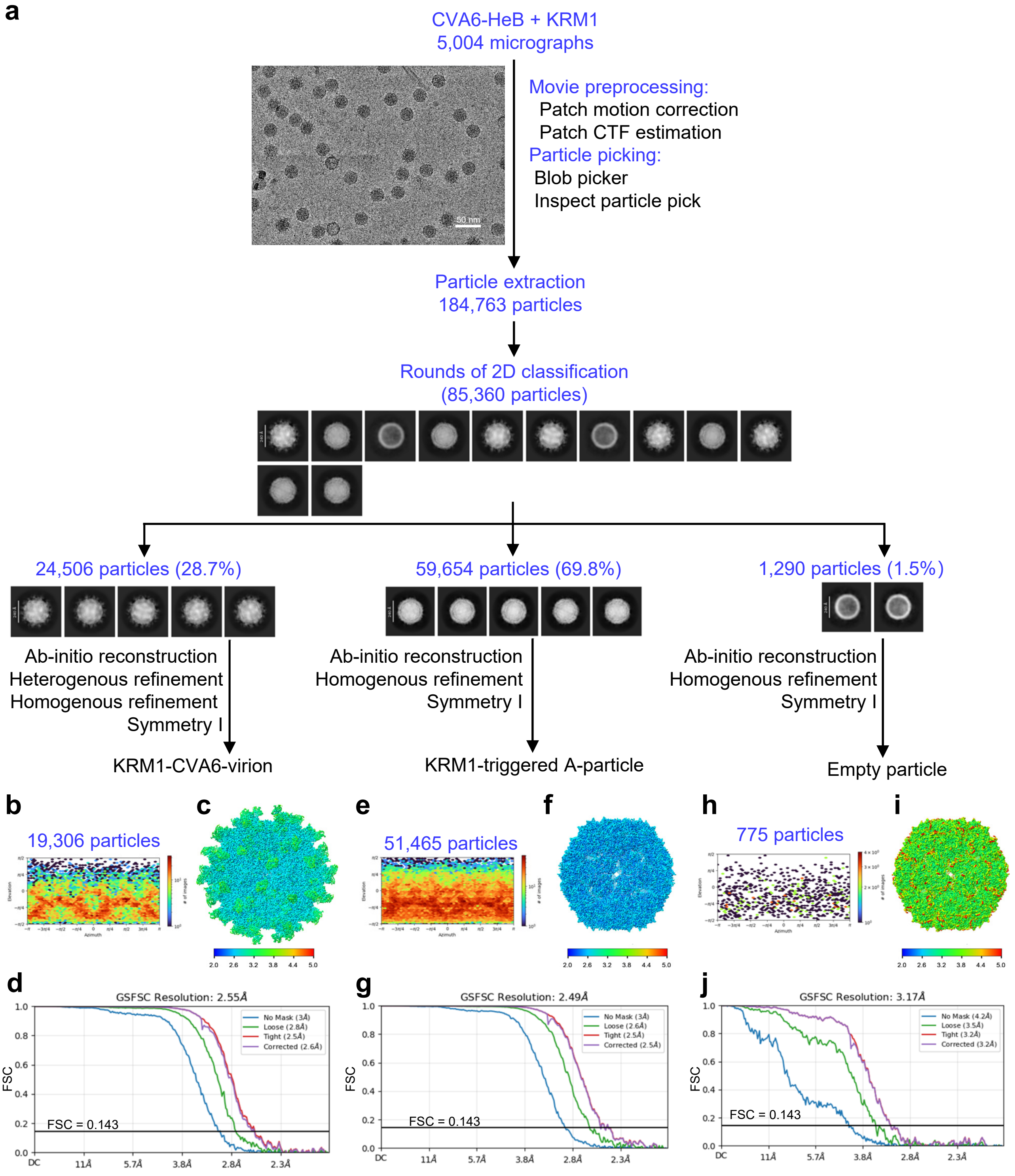

Supplementary Figure 6. Cryo-EM structural analysis of KRM1-treated CVA6 particles. (**a**) Representative cryo-EM micrograph of KRM1-treated CVA6-HeB viral particles and the corresponding data processing workflow implemented in cryoSPARC. **(b, c, d)** Particle angular distribution, local, and global resolution estimation of the KRM1-bound CVA6 virion. **(e, f, g)** Particle angular distribution, local, and global resolution estimation of the KRM1-triggered A-particle. **(h, i, j)** Particle angular distribution, local, and global resolution estimation of empty particle. Local resolutions were calculated in cryoSPARC (v4.5.3) and visualized in UCSF ChimeraX (v1.9). Global resolutions were determined in cryoSPARC based on the gold-standard FSC curve at a cutoff of 0.143.

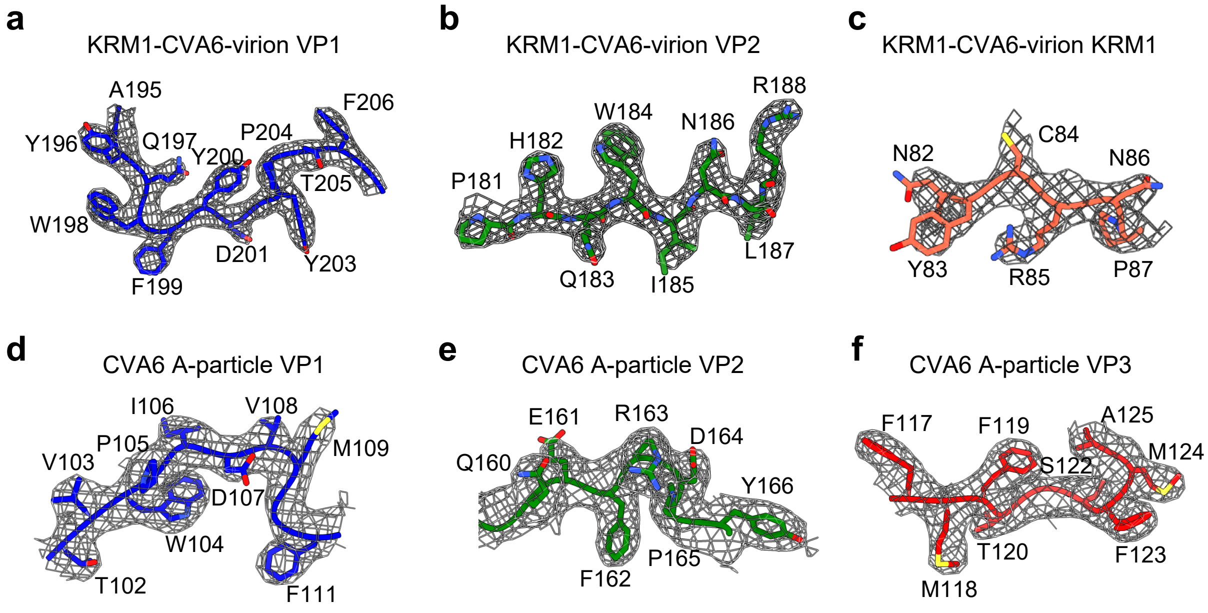

Supplementary Figure 7. Quality of the cryo-EM density maps for the CVA6-KRM1 complex and A-particle (related to **Figure 3i-k**). Representative structural motifs (colored sticks) are shown fitted into their corresponding density maps (gray mesh) for the KRM1-bound CVA6 virion (**a-c**, VP1/blue, VP2/green, KRM1/tomato) and the CVA6 A-particle (**d-f**, VP1/blue, VP2/green, VP3/red).

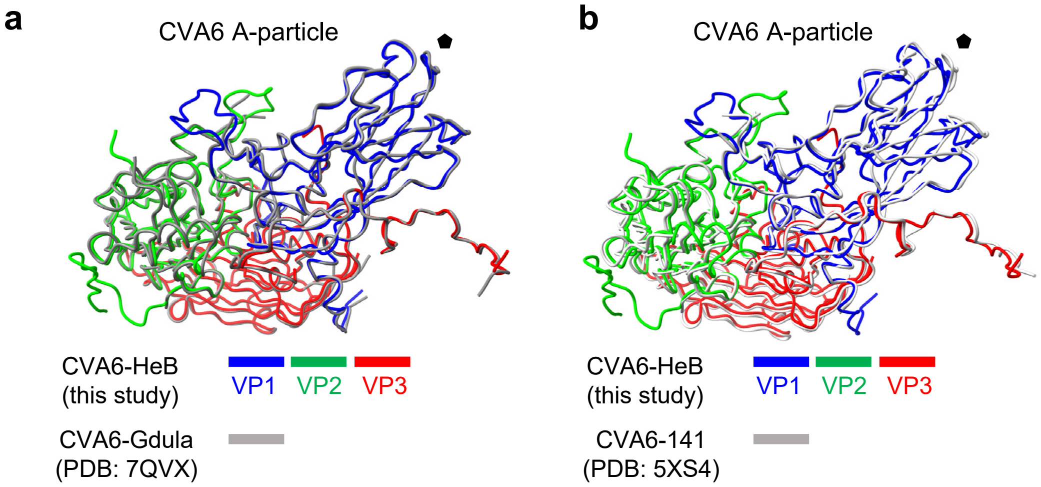

c

A-particle		VP1	VP2	VP3	Protomer
CVA6-HeB (this study) vs. CVA6-Gdula (PDB: 7QVX)	Aligned Cα	219 / 219	215 / 217	217 / 220	651 / 658
	RMSD (Å)	0.4	0.4	0.4	0.5
CVA6-HeB (this study) vs. CVA6-141 (PDB: 5XS4)	Aligned Cα	212 / 212	203 / 203	204 / 210	619 / 626
	RMSD (Å)	0.7	0.6	0.8	1.0

Supplementary Figure 8. Structural comparison of CVA6 A-particle protomers across strains. Note that CVA6-HeB A-particles were generated via KRM1-treated mature virions, while Gdula and 141 strain A-particles were derived from standard virus purification. (**a**) Superposition of A-particle protomers from the HeB strain (colored) and the Gdula strain (gray; PDB: 7QVX), shown as ribbon diagrams. CVA6-HeB subunits are labeled: VP1 (blue), VP2 (green), and VP3 (red). (**b**) Structural alignment of protomer of CVA6-HeB A-particle (colored) with 141 strain A-particle (gray; PDB: 5XS4). (**c**) RMSD analysis of A-particle protomer of CVA6-HeB against Gdula (PDB: 7QVX) and 141 (PDB: 5XS4) strains. Structural alignments and RMSD calculations were performed in UCSF ChimeraX. Protomer chains were preprocessed with WinCoot.

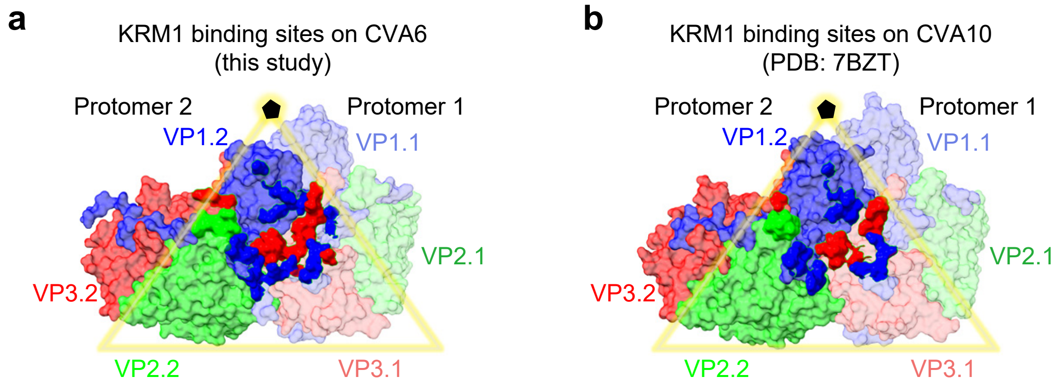

c

VP1.1 — C-terminus

```
                270            280           290
CVA6    AYMVKNYPTY-SQTITNTATDRASITTTDY------EGGV
CVA10   PYLLKNFPNYDSSKITNSARDRSSIKQANMGKFGQQSGAI
```

VP3.1 — AB-loop / CD-loop / GH-loop / C-terminus

```
        AB-loop        CD-loop                  GH-loop        C-terminus
          60            90          100           180            230
CVA6    VNNTTGTTGVS    QVDPGRNGPWQSTMVGQICRY    AVKTGGVYDYY    KDTDEIQQTAEYQ
CVA10   VNNTTEATGLT    MVDPGRIGPWQSTLVGQICRY    TAKTGGNYDYY    KDTDEVTQQAVLQ
```

VP1.2 — BC-loop / EF-loop / GH-loop

```
        BC-loop        EF-loop             GH-loop
          100           160        170       210        220
CVA6    TSLDGYTVWPI    KPDSRKSYQWQTAT     TFGEHKQATNLQYGQCPNNMMGH
CVA10   TDTTGYATWDI    KPTGRDAFQWQTAT     TFGQHPETSNTTYGLCPNNMMGT
```

VP2.2 — EF-loop

```
               140
CVA6    AASSPATKPNSQGLYP
CVA10   AGRGSNTKPNEAPH-P
```

VP3.2 — C-terminus

```
         230
CVA6    KDTDEIQQTAEYQ
CVA10   KDTDEVTQQAVLQ
```

Supplementary Figure 9. Comparative analysis of KRM1 binding sites in CVA6 and CVA10. (**a**) KRM1 binding sites (highlighted in deep blue for VP1, deep green for VP2, deep red for VP3) mapped onto two adjacent CVA6 protomers. Capsid subunits are shown as ribbons: Protomer 1 (VP1.1 light blue, VP2.1 light green, VP3.1 light red); Protomer 2 (VP1.2 medium blue, VP2.2 medium green, VP3.2 medium red). An icosahedral asymmetric unit is marked by a triangle. (**b**) KRM1 binding sites (colored as in panel A) on adjacent CVA10 protomers. Subunit coloring matches panel A. (**c**) Sequence alignment of KRM1-interacting regions in CVA6 and CVA10, with secondary structure elements labeled above. Interacting residues are annotated: hydrogen bonds, salt bridges, or π–cation interactions (magenta); PDBePISA analysis-identified contacts (<4 Å, cyan). VP2 residue K140, critical for CVA6 and CVA10 binding to KRM1, is indicated by a red arrow.

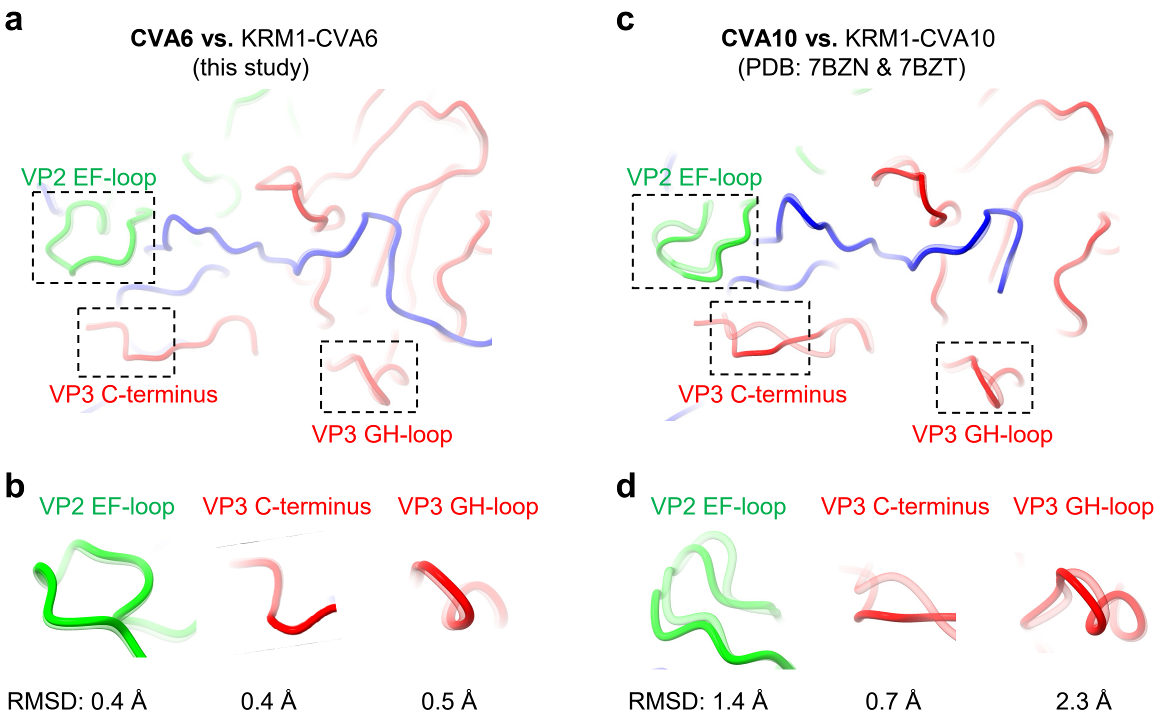

Supplementary Figure 10. Structural comparison of CVA6 and CVA10 particles with and without KRM1 binding. (**a**) Superposition of the icosahedral asymmetric units of CVA6 with and without KRM1 (unbound: dark colors; KRM1-bound: light colors), showing minor KRM1-induced conformational changes. KRM1 is omitted for clarity. (**b**) Zoomed views of boxed regions in panel (**a**): VP2 EF-loop, VP3 C terminus, and VP3 GH-loop rearrangements with calculated RMSD values. (**c**) CVA10 structural comparison (unbound [PDB: 7BZN] vs. KRM1-bound [PDB: 7BZT]), highlighting conformational changes upon KRM1 binding. Color scheme matches (**a**). (**d**) Enlarged views of boxed regions from panel (**c**) showing local structural differences and RMSD values.

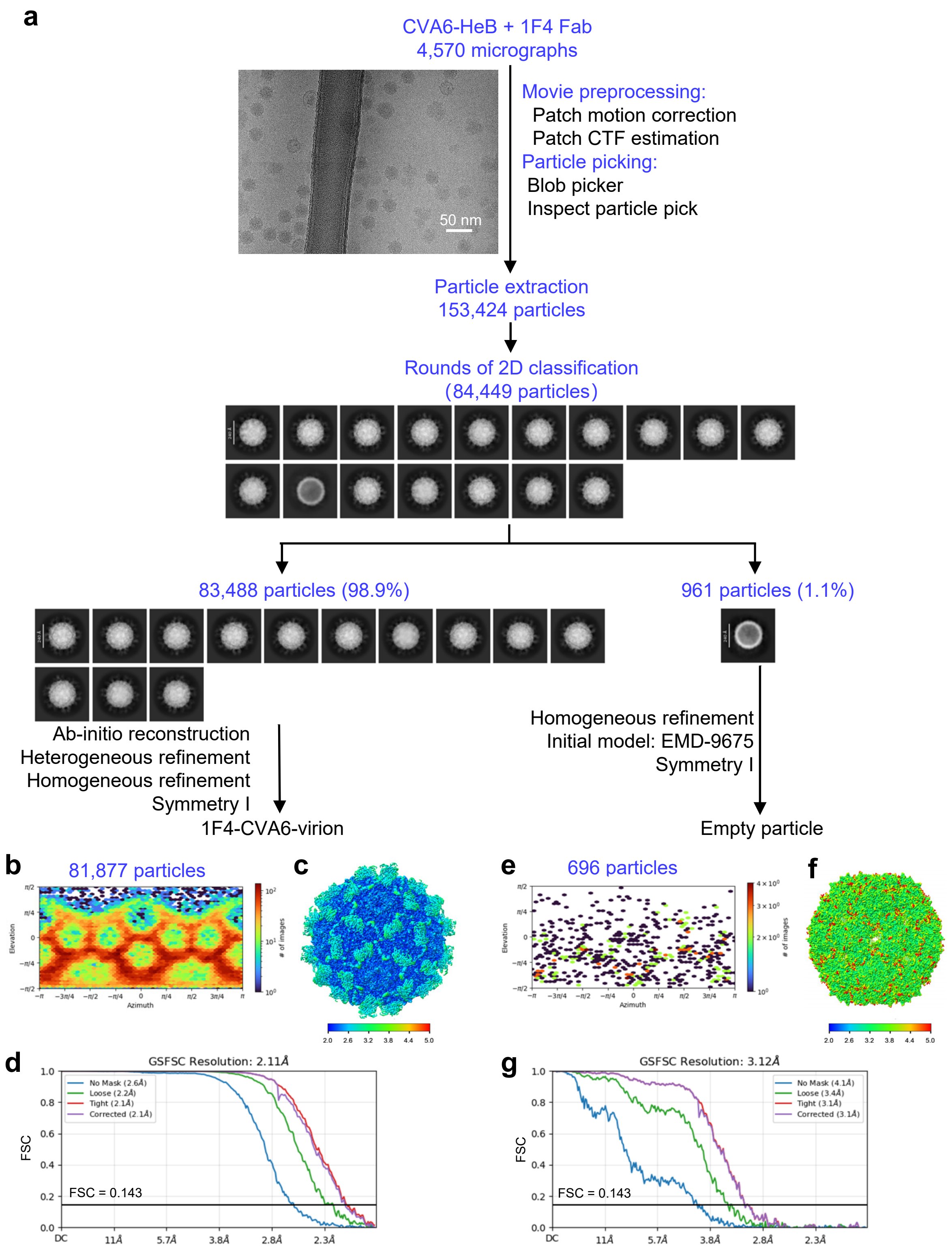

Supplementary Figure 11. Cryo-EM structural analysis of CVA6 particles in complex with 1F4 Fab. (**a**) Representative cryo-EM micrograph of CVA6-HeB viral particles complexed with 1F4 Fab and the corresponding data processing workflow executed in cryoSPARC. The 3D reconstruction of CVA6 empty particle was generated using a homology modeling strategy with the reported CVA10 empty particle (EMD-9675) as the initial reference. **(b, c, d)** Particle angular distribution, local, and global resolution estimation of 1F4-bound CVA6 virion. **(e, f, g)** Particle angular distribution, local, and global resolution estimation of empty particle. Local resolutions were calculated in cryoSPARC (v4.5.3) and visualized in UCSF ChimeraX (v1.9). Global resolutions were determined in cryoSPARC based on the gold-standard FSC curve at a cutoff of 0.143.

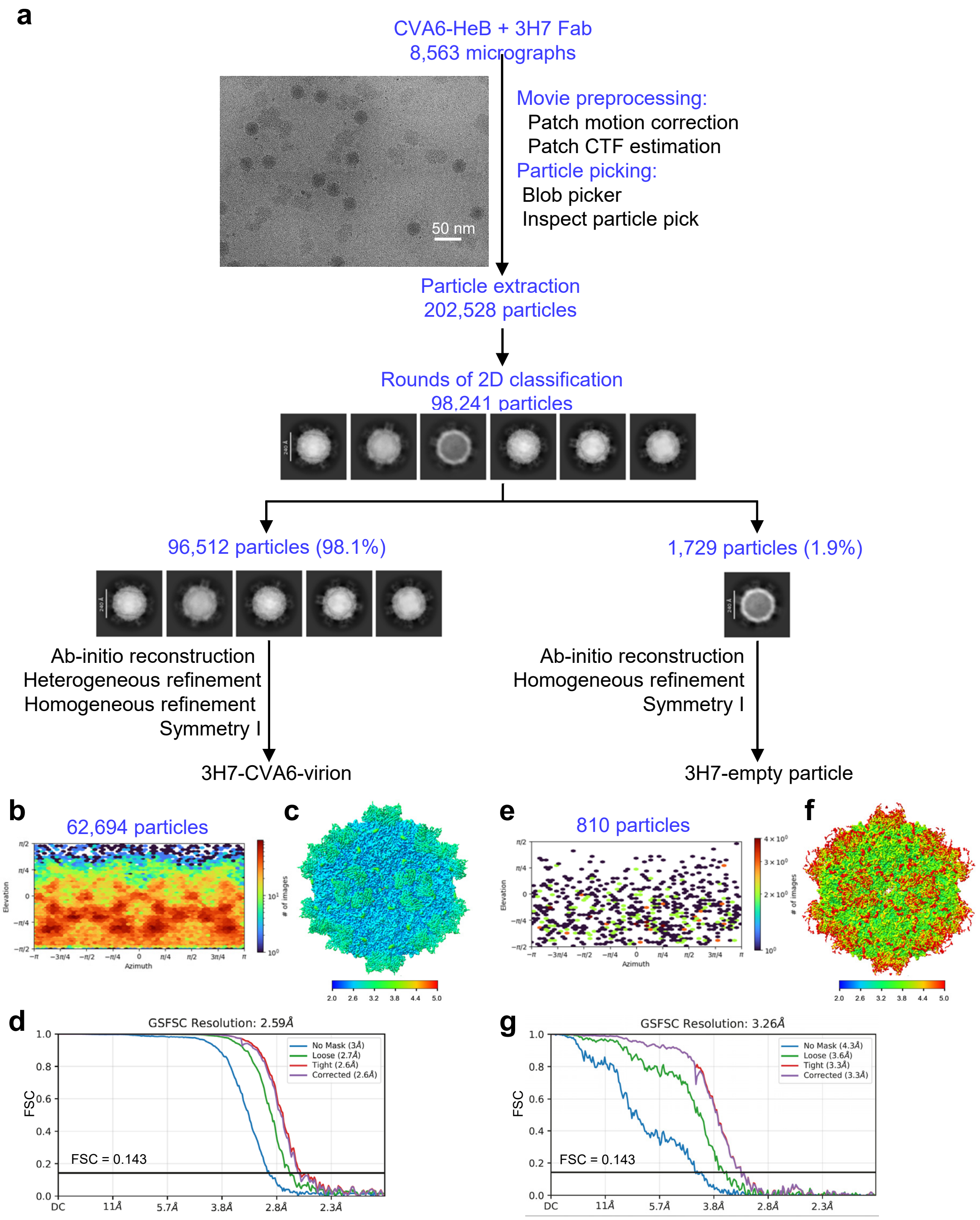

Supplementary Figure 12. Cryo-EM structural analysis of CVA6-3H7 Fab complexes. (**a**) Representative cryo-EM micrograph of 3H7 Fab-bound CVA6-HeB viral particles and the data processing workflow in cryoSPARC. Reconstructed maps were radially color-coded using UCSF ChimeraX. **(b, c, d)** Particle angular distribution, local, and global resolution estimation of 3H7-bound mature CVA6 virion. **(e, f, g)** Particle angular distribution, local, and global resolution estimation of 3H7-associated empty particle. Local resolutions were calculated in cryoSPARC (v4.5.3) and visualized in UCSF ChimeraX (v1.9). Global resolutions were determined in cryoSPARC based on the gold-standard FSC curve at a cutoff of 0.143.

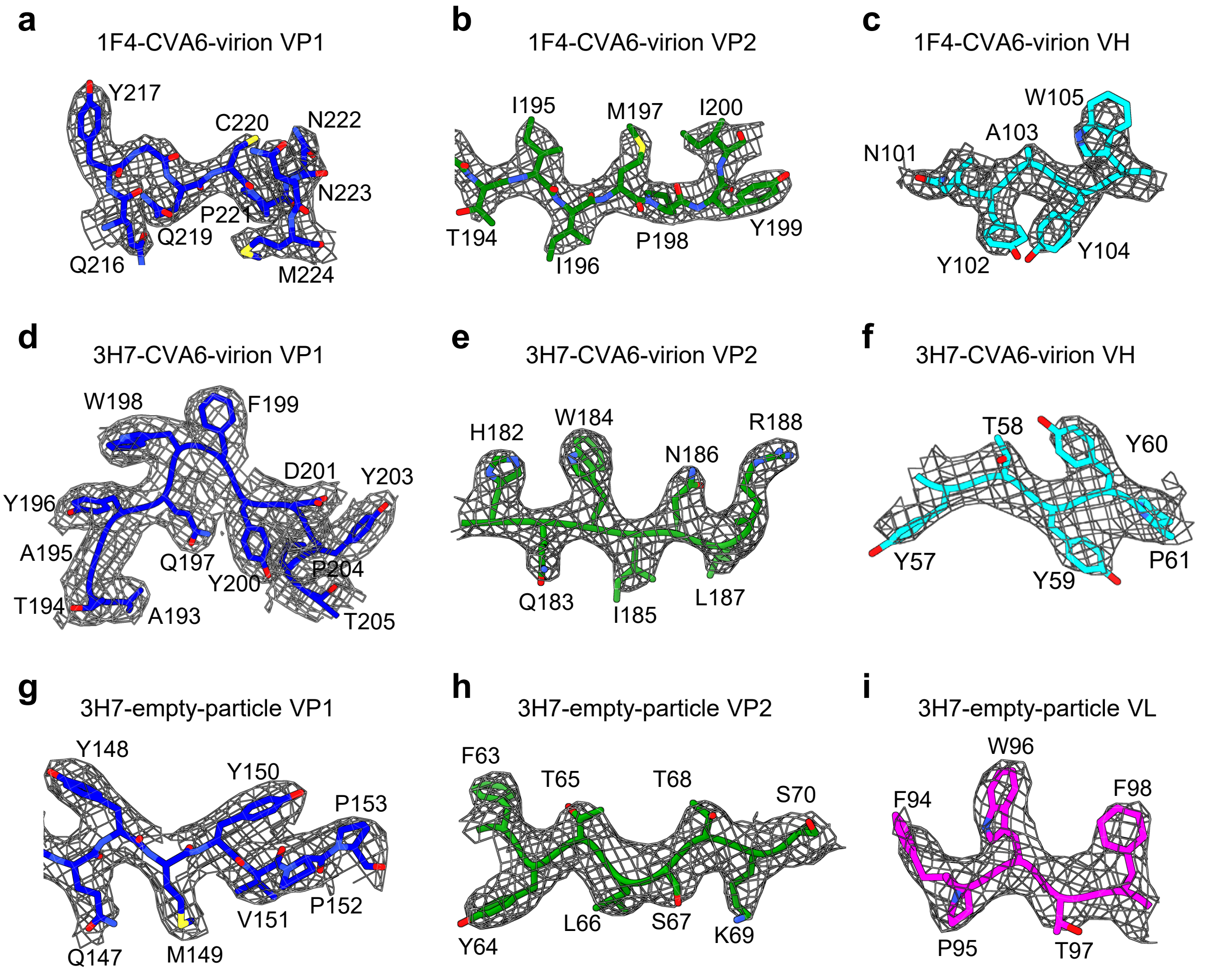

Supplementary Figure 13. Quality of the cryo-EM density maps for CVA6-antibody complexes (related to **Figures 5–6**). Representative structural motifs (colored sticks) are shown fitted into their corresponding density maps (gray mesh). 1F4-CVA6-virion complex: (**a**) VP1 (blue), (**b**) VP2 (green), (**c**) 1F4 VH domain (cyan). 3H7-CVA6-virion complex: (**d**) VP1 (blue), (**e**) VP2 (green), (**f**) 3H7 VH domain (cyan). 3H7-empty particle: (**g**) VP1 (blue), (**h**) VP2 (green), (**i**) 3H7 VL domain (magenta).

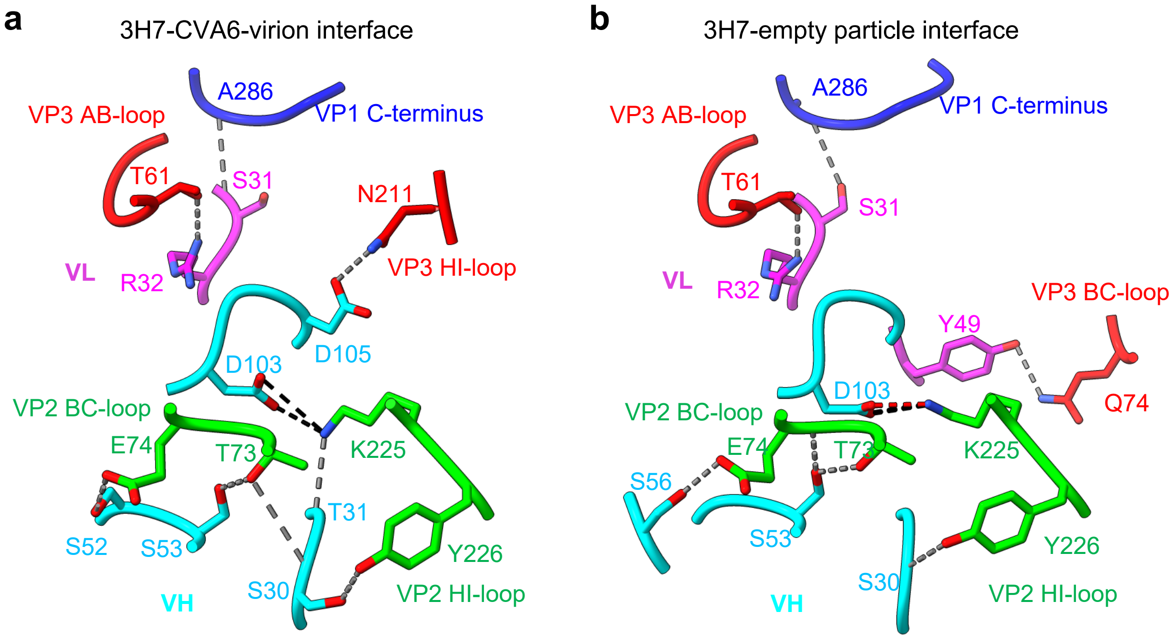

Supplementary Figure 14. Comparative interface analysis of 3H7 Fab interactions with CVA6 mature virion and empty particle. (**a**) Molecular interface analysis between 3H7 variable domains (VH: cyan; VL: magenta) and CVA6 mature virion. (**b**) Interface analysis of 3H7 Fab binding to CVA6 empty particle. Viral proteins are color-coded as VP1 (blue), VP2 (green), and VP3 (red). Gray dashed lines indicate hydrogen bonds, black dashed lines denote salt bridges, and red dashed lines represent interactions that are both hydrogen bonds and salt bridges.

a

MAb	Neutralization conc. (μg/ml)						
	CVA6-141	CVA2-HN	CVA3-XZ	CVA4-FJ	CVA5-ZZ285	CVA8-GS14	CVA12-Texas
1F4	0.156	>10	>10	>10	>10	>10	>10
3H7	0.313	>10	>10	>10	>10	>10	>10

b

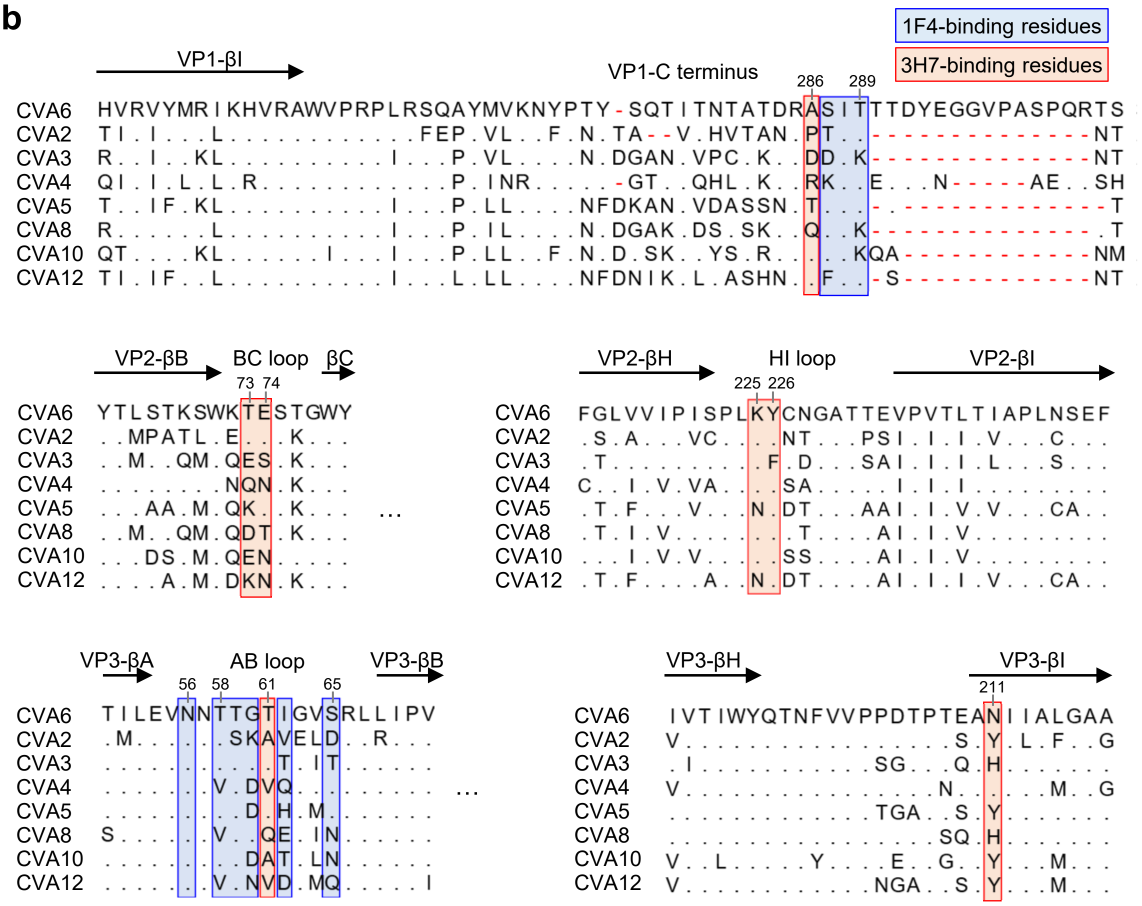

Supplementary Figure 15. Specificity of CVA6-neutralizing MAbs 1F4 and 3H7. (**a**) MAbs 1F4 and 3H7 show no neutralization activity against a panel of related KRM1-using enteroviruses at concentrations up to 10 μg/mL, demonstrating they are CVA6-specific. (**b**) Sequence alignment reveals the molecular basis for specificity: key binding residues for 1F4 and 3H7 (colored) are not conserved across the capsid proteins of CVA6 (strain 141), CVA2 (HN), CVA3 (XZ), CVA4 (FJ), CVA5 (ZZ285), CVA8 (GS14), CVA10 (S0148b), and CVA12 (Texas). Dots represent residues identical to those of CVA6, and red dashes are gaps.

Supplementary Table 1. Cryo-EM data collection and refinement statistics for CVA6 particles.

	**CVA6-HeB virion** (EMD-60531) (PDB: 9VFQ)	**CVA6-HeB empty particle** (EMD-65030) (PDB: 9VFP)
**Data collection and processing**		
Magnification	75,000	75,000
Voltage (keV)	300	300
Pixel size (Å)	1.05	1.05
Defocus	1.0-2.0	1.0-2.0
Symmetry imposed	I	I
Map resolution (Å)	2.52	3.47
FSC threshold	0.143	0.143
Map sharpening B factor ($Å^2$)	-84	-55.7
**Model composition**		
Non-hydrogen atoms	6270	4880
Protein residues	826	640
Water	0	0
**RMS deviation from ideality**		
Bond lengths (Å)	0.007	0.010
Bond angles (º)	0.58	0.710
**Validation**		
MolProbity score	1.46	1.74
Clash score	5.21	4.91
Rotamer outliers (%)	0.46	0.58
**Ramachandran statistics**		
Favored regions (%)	96.94	92.01
Allowed regions (%)	3.06	7.99
Outlier (%)	0	0

Supplementary Table 2. Cryo-EM data collection and refinement statistics for KRM1-treated CVA6 particles.

	**KRM1-CVA6-virion** (EMD-65032) (PDB: 9VFR)	**A-particle** (EMD-65034) (PDB: 9VFS)
**Data collection and processing**		
Magnification	50,000	50,000
Voltage (keV)	300	300
Pixel size (Å)	0.95	0.95
Defocus	1.0-2.0	1.0-2.0
Symmetry imposed	I	I
Map resolution (Å)	2.55	2.49
FSC threshold	0.143	0.143
Map sharpening B factor (Å2)	-81.8	-87.8
**Model composition**		
Non-hydrogen atoms	14803	5379
Protein residues	1945	695
Water	0	0
**RMS deviation from ideality**		
Bond lengths (Å)	0.007	0.007
Bond angles (°)	0.519	0.575
**Validation**		
MolProbity score	1.79	2.02
Clash score	8.11	8.31
Rotamer outliers (%)	1.18	1.86
**Ramachandran statistics**		
Favored regions (%)	95.80	94.76
Allowed regions (%)	4.20	5.24
Outlier (%)	0	0

Supplementary Table 3. Interaction interface analysis of the KRM1-CVA6-virion structure.

CVA6			Distance (Å)	KRM1		Interaction
Protomer	Location	Residue		Residue	Location	
Protomer 1	VP1 C-terminus	D284 [O]	3.22	T135 [OG1]	WSC	H-bond
		D284 [O]	3.27	S136 [N]		H-bond
		A286 [N]	3.27	T135 [OG1]		H-bond
		D292 [OD2]	2.82	T138 [N]		H-bond
		D292 [OD2]	3.29	T138 [OG1]		H-bond
		E294 [OE1]	3.29	N140 [N]		H-bond
		E294 [OE1]	3.28	K141 [N]		H-bond
		E294 [OE2]	3.43	K141 [N]		H-bond
		E294 [OE2]	2.22	S139 [OG]		H-bond
	VP3 CD-loop	R90 [NH1]	3.01	T138 [OG1]	WSC	H-bond
	VP3 C-terminus	D231 [O]	2.98	N128 [ND2]		H-bond
		Q234 [NE2]	3.13	Y178 [OH]		H-bond
Protomer 2	VP1 EF-loop	K157 [NZ]	2.71	D201 [OD2]	WSC	H-bond, salt bridge
		D159 [OD2]	2.54	H126[ND1]		H-bond, salt bridge
		D159 [OD2]	3.73	H126[NE2]		Salt bridge
	VP1 GH-loop	Q211 [NE2]	2.93	G192 [O]		H-bond
	VP2 EF-loop	K140 [NZ]	3.09	D90 [OD2]	KR	H-bond, salt bridge
		K140 [NZ]	2.49	D88 [OD2]		Salt bridge
		K140 [NZ]	3.75	D88 [OD1]		Salt bridge
		N142 [ND2]	3.39	G89 [O]		H-bond
		N142 [ND2]	3.45	D90 [OD1]		H-bond

Supplementary Table 4. Cryo-EM data collection and refinement statistics for 1F4 Fab-treated CVA6 particles.

	**1F4-CVA6-virion** (EMD-65038) (PDB: 9VFU)
**Data collection and processing**	
Magnification	50,000
Voltage (keV)	300
Pixel size (Å)	0.95
Defocus	1.0-2.0
Symmetry imposed	I
Map resolution (Å)	2.11
FSC threshold	0.143
Map sharpening B factor (Å2)	-68.9
**Model composition**	
Non-hydrogen atoms	7733
Protein residues	1036
Water	0
**RMS deviation from ideality**	
Bond lengths (Å)	0.007
Bond angles (º)	0.542
**Validation**	
MolProbity score	1.65
Clash score	6.74
Rotamer outliers (%)	0.65
**Ramachandran statistics**	
Favored regions (%)	95.99
Allowed regions (%)	4.01
Outlier (%)	0

Supplementary Table 5. Interaction interface analysis of the 1F4-CVA6-virion structure.

CVA6 mature virion		Distance (Å)	1F4		Interaction
Location	Residue		Residue	Location	
VP1 C-terminus	S287 [OG]	2.97	N53 [ND2]	VL FR3	H-bond
	T289 [OG1]	3.28	N53 [ND2]		H-bond
	I288 [N]	2.76	N101 [OD1]	VH CDR3	H-bond
VP3 AB-loop (knob)	N56 [OD1]	2.94	N101 [ND2]	VH CDR3	H-bond
	T58 [OG1]	3.27	N101 [ND2]	VH CDR3	H-bond
	G60 [O]	2.94	Y33 [N]	VH CDR1	H-bond
	S65 [OG]	2.73	S31 [OG]		H-bond
	T62 [N]	2.77	S31 [O]		H-bond
	T62 [OG1]	3.34	T30 [O]		H-bond
	T59 [OG1]	3.31	N32 [OD1]	VL CDR1	H-bond

Supplementary Table 6. Surface area of CVA6 viral capsid covered by 1F4 and 3H7 (in Fab-CVA6-virion structure) determined using PISA.

MAb	Interface area (Å^2)	
	Total (Å^2)	Contribution
1F4-VH	520.8	61.8%
1F4-VL	321.6	38.2%
1F4	842.4	100%
3H7-VH	787.9	70.1%
3H7-VL	336.5	29.9%
3H7	1124.4	100%

Supplementary Table 7. Cryo-EM data collection and refinement statistics for 3H7 Fab-treated CVA6 particles.

	**3H7-CVA6-virion** (EMD-65036) (PDB: 9VFT)	**3H7-empty particle** (EMD-65043) (PDB: 9VG1)
**Data collection and processing**		
Magnification	50,000	50,000
Voltage (keV)	300	300
Pixel size (Å)	0.95	0.95
Defocus	1.0-2.0	1.0-2.0
Symmetry imposed	I	I
Map resolution (Å)	2.59	3.26
FSC threshold	0.143	0.143
Map sharpening B factor (Å2)	-109.0	-54.6
**Model composition**		
Non-hydrogen atoms	7649	6443
Protein residues	1053	866
Water	0	0
**RMS deviation from ideality**		
Bond lengths (Å)	0.005	0.003
Bond angles (°)	0.518	0.525
**Validation**		
MolProbity score	1.57	2.10
Clash score	4.65	8.36
Rotamer outliers (%)	0.86	1.70
**Ramachandran statistics**		
Favored regions (%)	95.19	92.45
Allowed regions (%)	4.81	7.55
Outlier (%)	0	0

Supplementary Table 8. Interaction interface analysis of the 3H7-CVA6-virion structure (upper table) and the 3H7-empty particle structure (lower table).

<table>
<tr><th colspan="2">CVA6 mature virion</th><th rowspan="2">Distance (Å)</th><th colspan="2">3H7</th><th rowspan="2">Interaction</th></tr>
<tr><th>Location</th><th>Residue</th><th>Residue</th><th>Location</th></tr>
<tr><td>VP1 C-terminus</td><td>A286 [O]</td><td>3.16</td><td>S31 [N]</td><td>VL CDR1</td><td>H-bond</td></tr>
<tr><td rowspan="3">VP2 BC-loop</td><td>T73 [OG1]</td><td>3.50</td><td>S30 [O]</td><td>VH CDR1</td><td>H-bond</td></tr>
<tr><td>T73 [OG1]</td><td>2.20</td><td>S53 [OG]</td><td>VH CDR2</td><td>H-bond</td></tr>
<tr><td>E74 [OE1]</td><td>3.19</td><td>S52 [OG]</td><td>VH CDR2</td><td>H-bond</td></tr>
<tr><td rowspan="4">VP2 HI-loop</td><td>K225 [NZ]</td><td>2.36</td><td>T31 [O]</td><td>VH CDR1</td><td>H-bond</td></tr>
<tr><td>K225 [NZ]</td><td>4.00</td><td>D103 [OD1]</td><td>VH CDR3</td><td>Salt bridge</td></tr>
<tr><td>K225 [NZ]</td><td>2.93</td><td>D103 [OD2]</td><td>VH CDR3</td><td>Salt bridge</td></tr>
<tr><td>Y 226 [OH]</td><td>2.35</td><td>S30 [OG]</td><td>VH CDR1</td><td>H-bond</td></tr>
<tr><td>VP3 AB-loop</td><td>T61 [OG1]</td><td>2.93</td><td>R 32 [NH1]</td><td>VL CDR1</td><td>H-bond</td></tr>
<tr><td>VP3 βI</td><td>N211 [ND2]</td><td>2.49</td><td>D105 [OD1]</td><td>VH CDR3</td><td>H-bond</td></tr>
</table>

<table>
<tr><th colspan="2">CVA6 empty particle</th><th rowspan="2">Distance (Å)</th><th colspan="2">3H7</th><th rowspan="2">Interaction</th></tr>
<tr><th>Location</th><th>Residue</th><th>Residue</th><th>Location</th></tr>
<tr><td>VP1 C-terminus</td><td>A286 [O]</td><td>3.43</td><td>S31 [OG]</td><td>VL CDR1</td><td>H-bond</td></tr>
<tr><td rowspan="3">VP2 BC-loop</td><td>T73 [OG1]</td><td>2.38</td><td>S53 [OG]</td><td rowspan="3">VH CDR2</td><td>H-bond</td></tr>
<tr><td>E74 [N]</td><td>3.22</td><td>S53 [OG]</td><td>H-bond</td></tr>
<tr><td>E74 [OE1]</td><td>2.41</td><td>S56 [OG]</td><td>H-bond</td></tr>
<tr><td rowspan="3">VP2 HI-loop</td><td>K225 [NZ]</td><td>2.70</td><td>D103 [OD1]</td><td rowspan="2">VH CDR3</td><td>H-bond, salt bridge</td></tr>
<tr><td>K225 [NZ]</td><td>3.45</td><td>D103 [OD2]</td><td>Salt bridge</td></tr>
<tr><td>Y226 [OH]</td><td>3.04</td><td>S30 [O]</td><td>VH CDR1</td><td>H-bond</td></tr>
<tr><td>VP3 AB-loop</td><td>T61 [OG1]</td><td>2.73</td><td>R32 [NH1]</td><td>VL CDR1</td><td>H-bond</td></tr>
<tr><td>VP3 BC-loop</td><td>Q74 [NE2]</td><td>3.28</td><td>Y49 [OH]</td><td>VL FR2</td><td>H-bond</td></tr>
</table>
