# Supplementary material for: Molecular mechanisms of receptor recognition and antibody neutralization of coxsackievirus A6
Source: Nat Commun. 2025 Dec 18;17:934. doi: 10.1038/s41467-025-67666-9 (PMC12830800; doi:10.1038/s41467-025-67666-9)
Supplement: Supplementary file 3 — Reporting Summary [file 41467_2025_67666_MOESM3_ESM.pdf]

nature portfolio

Corresponding author(s): Chao Zhang

Last updated by author(s): November 30th, 2025

Reporting Summary

Nature Portfolio wishes to improve the reproducibility of the work that we publish. This form provides structure for consistency and transparency in reporting. For further information on Nature Portfolio policies, see our Editorial Policies and the Editorial Policy Checklist.

Statistics

For all statistical analyses, confirm that the following items are present in the figure legend, table legend, main text, or Methods section.

n/a	Confirmed	
☐	☒	The exact sample size (*n*) for each experimental group/condition, given as a discrete number and unit of measurement
☐	☐	A statement on whether measurements were taken from distinct samples or whether the same sample was measured repeatedly
☐	☒	The statistical test(s) used AND whether they are one- or two-sided<br>*Only common tests should be described solely by name; describe more complex techniques in the Methods section.*
☒	☐	A description of all covariates tested
☒	☐	A description of any assumptions or corrections, such as tests of normality and adjustment for multiple comparisons
☐	☒	A full description of the statistical parameters including central tendency (e.g. means) or other basic estimates (e.g. regression coefficient) AND variation (e.g. standard deviation) or associated estimates of uncertainty (e.g. confidence intervals)
☐	☒	For null hypothesis testing, the test statistic (e.g. *F*, *t*, *r*) with confidence intervals, effect sizes, degrees of freedom and *P* value noted<br>*Give P values as exact values whenever suitable.*
☒	☐	For Bayesian analysis, information on the choice of priors and Markov chain Monte Carlo settings
☒	☐	For hierarchical and complex designs, identification of the appropriate level for tests and full reporting of outcomes
☒	☐	Estimates of effect sizes (e.g. Cohen's *d*, Pearson's *r*), indicating how they were calculated

Our web collection on statistics for biologists contains articles on many of the points above.

Software and code

Policy information about availability of computer code

Data collection	SerialEM
Data analysis	GraphPad Prism version 8, CryoSPARC v4.5.3, Phenix 1.17.1, WinCoot 0.9.8.95, UCSF Chimera 1.1.8, UCSF ChimeraX 1.9, PDBePISA server, RIVEM v4.5

For manuscripts utilizing custom algorithms or software that are central to the research but not yet described in published literature, software must be made available to editors and reviewers. We strongly encourage code deposition in a community repository (e.g. GitHub). See the Nature Portfolio guidelines for submitting code & software for further information.

Data

Policy information about availability of data

All manuscripts must include a data availability statement. This statement should provide the following information, where applicable:

- Accession codes, unique identifiers, or web links for publicly available datasets
- A description of any restrictions on data availability
- For clinical datasets or third party data, please ensure that the statement adheres to our policy

The atomic coordinates of the CVA6 mature virion, CVA6 empty particles, KRM1-CVA6-virion complex, KRM1-triggered CVA6 A-particle, 1F4-CVA6-virion complex, 3H7-CVA6-virion complex, and 3H7-CVA6 empty particle complex have been deposited in the Protein Data Bank (PDB) under accession codes 9VFQ, 9VFP, 9VFR, 9VFS, 9VFU, 9VFT and 9VG1, respectively. The corresponding unsharpened cryo-EM density maps have been deposited in the Electron Microscopy Data Bank (EMDB) under accession codes EMD-65031, EMD-65030, EMD-65032, EMD-65034, EMD-65038, EMD-65036 and EMD-65043, respectively. The sequences of 1F4-

VH, 1F4-VL, 3H7-VH, and 3H7-VL have been deposited in DNA Data Bank of Japan (DDBJ) under accession codes LC900916, LC900917, LC900918, and LC901458, respectively. Source data are provided with this paper.

Research involving human participants, their data, or biological material

Policy information about studies with human participants or human data. See also policy information about sex, gender (identity/presentation), and sexual orientation and race, ethnicity and racism.

Reporting on sex and gender	Our study did not involve human participants, data, or biological specimens.
Reporting on race, ethnicity, or other socially relevant groupings	*Please specify the socially constructed or socially relevant categorization variable(s) used in your manuscript and explain why they were used. Please note that such variables should not be used as proxies for other socially constructed/relevant variables (for example, race or ethnicity should not be used as a proxy for socioeconomic status).* *Provide clear definitions of the relevant terms used, how they were provided (by the participants/respondents, the researchers, or third parties), and the method(s) used to classify people into the different categories (e.g. self-report, census or administrative data, social media data, etc.)* *Please provide details about how you controlled for confounding variables in your analyses.*
Population characteristics	*Describe the covariate-relevant population characteristics of the human research participants (e.g. age, genotypic information, past and current diagnosis and treatment categories). If you filled out the behavioural & social sciences study design questions and have nothing to add here, write "See above."*
Recruitment	*Describe how participants were recruited. Outline any potential self-selection bias or other biases that may be present and how these are likely to impact results.*
Ethics oversight	*Identify the organization(s) that approved the study protocol.*

Note that full information on the approval of the study protocol must also be provided in the manuscript.

Field-specific reporting

Please select the one below that is the best fit for your research. If you are not sure, read the appropriate sections before making your selection.

☒ Life sciences ☐ Behavioural & social sciences ☐ Ecological, evolutionary & environmental sciences

For a reference copy of the document with all sections, see nature.com/documents/nr-reporting-summary-flat.pdf

Life sciences study design

All studies must disclose on these points even when the disclosure is negative.

Sample size	For animal experiments, each group included 11–16 mice. The sample size was sufficient for a good statistical analysis.
Data exclusions	No data were excluded from the analysis.
Replication	Experimental findings were reliably reproduced. Most of the experiments were replicated two or three times.
Randomization	Animals were randomly divided into experimental groups.
Blinding	No blinding is used, because the primary outcomes measured were objective quantitative parameters. Therefore, the risk of assessment bias was considered minimal.

Reporting for specific materials, systems and methods

We require information from authors about some types of materials, experimental systems and methods used in many studies. Here, indicate whether each material, system or method listed is relevant to your study. If you are not sure if a list item applies to your research, read the appropriate section before selecting a response.

Materials & experimental systems

n/a	Involved in the study	
☐	☒	Antibodies
☐	☒	Eukaryotic cell lines
☒	☐	Palaeontology and archaeology
☐	☒	Animals and other organisms
☒	☐	Clinical data
☒	☐	Dual use research of concern
☒	☐	Plants

Methods

n/a	Involved in the study	
☒	☐	ChIP-seq
☒	☐	Flow cytometry
☒	☐	MRI-based neuroimaging

Antibodies

Antibodies used	Anti-CVA6 MAbs (1F4 and 3H7; two-fold serially diluted), anti-SARS-CoV-2 MAb 3A2 (isotype control; two-fold serially diluted), and mouse anti-CVA6-VP0 polyclonal antibody (1:1,000) were prepared in our lab. HRP-conjugated anti-mouse IgG (1:10,000; Cat: SA00001-1) and HRP-conjugated streptavidin (1:5,000; Cat: SA00001-0) were purchased from Proteintech (China).
Validation	The specifications of commercially available antibody can be found on the manufacture's website using their catalogue numbers. Anti-CVA6 MAbs 1F4 and 3H7 were prepared and validated in this study. Anti-SARS-CoV-2 MAb 3A2 was used as isotype control, obtained and validated in a previous study (Zhang et al. Nat Commun, 2021. PMID: 33431876). Mouse anti-CVA6-VP0 polyclonal antibody was generated and validated in a previous study (Shen et al. Vaccine, 2016. PMID: 27340093).

Eukaryotic cell lines

Policy information about cell lines and Sex and Gender in Research

Cell line source(s)	Human rhabdomyosarcoma (RD) cells were obtained from ATCC (Cat: CCL-136). ΔKRM1 RD cells were generated and validated in a previous study (Liu et al. mBio, 2025. PMID: 39817751). ΔSLC35B2 RD cells were obtained from Dr. Mingzhou Chen (Hubei University) and validated in a previous study (Guo et al. J Virol, 2022. PMID: 35420441). SP2/0 myeloma cells were purchased from the Cell Bank of the Chinese Academy of Sciences (Shanghai, China). HEK 293F suspension cells were purchased from thermo fisher.
Authentication	The cell lines were not authenticated further after purchase.
Mycoplasma contamination	Cell lines have not recently been tested for mycoplasma contamination
Commonly misidentified lines (See ICLAC register)	No commonly misidentified lines were used

Animals and other research organisms

Policy information about studies involving animals; ARRIVE guidelines recommended for reporting animal research, and Sex and Gender in Research

Laboratory animals	BALB/c mice and ICR mice. All mice were purchased from Vital River Laboratory Animal Technology company (China).
Wild animals	No wild animals were used.
Reporting on sex	Female adult BALB/c mice and ICR mice. Male and female neonatal ICR mice.
Field-collected samples	No
Ethics oversight	The mouse studies were approved by the Institutional Animal Care and Use Committee of Fudan University.

Note that full information on the approval of the study protocol must also be provided in the manuscript.

Plants

Seed stocks	*Report on the source of all seed stocks or other plant material used. If applicable, state the seed stock centre and catalogue number. If plant specimens were collected from the field, describe the collection location, date and sampling procedures.*
Novel plant genotypes	*Describe the methods by which all novel plant genotypes were produced. This includes those generated by transgenic approaches, gene editing, chemical/radiation-based mutagenesis and hybridization. For transgenic lines, describe the transformation method, the number of independent lines analyzed and the generation upon which experiments were performed. For gene-edited lines, describe the editor used, the endogenous sequence targeted for editing, the targeting guide RNA sequence (if applicable) and how the editor was applied.*
Authentication	*Describe any authentication procedures for each seed stock used or novel genotype generated. Describe any experiments used to assess the effect of a mutation and, where applicable, how potential secondary effects (e.g. second site T-DNA insertions, mosiacism, off-target gene editing) were examined.*
